# Author Correction: Peptide-based inflammation-responsive implant coating sequentially regulates bone regeneration to enhance interfacial osseointegration

**DOI:** 10.1038/s41467-025-59573-w

**Published:** 2025-05-20

**Authors:** Wei Zhou, Yang Liu, Xuan Nie, Chen Zhu, Liming Xiong, Jing Zhou, Wei Huang

**Affiliations:** 1 Department of Orthopaedics, The First Affiliated Hospital of USTC, Division of Life Sciences and Medicine, University of Science and Technology of China, Hefei, China; 2 Department of Orthopaedics, Union Hospital, Tongji Medical College, Huazhong University of Science and Technology, Wuhan, China; 3 Department of Pharmacy, The First Affiliated Hospital of USTC, Division of Life Sciences and Medicine, Anhui Provincial Key Laboratory of Precision Pharmaceutical Preparations and Clinical Pharmacy, University of Science and Technology of China, Hefei, China; 4 Department of Urology, The First Affiliated Hospital of USTC, Division of Life Sciences and Medicine, University of Science and Technology of China, Hefei, China

**Keywords:** Translational research, Peptides

Correction to: *Nature Communications* 10.1038/s41467-025-58444-8, published online 6 April 2025

In the version of the article initially published, in Fig. 9a, the text “Day 0” appeared originally as “0” and “Week 8” appeared originally as “Week”; the figure is amended in the HTML and PDF versions of the article.

