# Supplementary material for: Peptide-based inflammation-responsive implant coating sequentially regulates bone regeneration to enhance interfacial osseointegration
Source: Nat Commun. 2025 Apr 6;16:3283. doi: 10.1038/s41467-025-58444-8 (PMC11973180; doi:10.1038/s41467-025-58444-8)
Supplement: Supplementary file 1 — Supplementary Information [file 41467_2025_58444_MOESM1_ESM.pdf]

Supplementary Information

Peptide-Based Inflammation-Responsive Implant Coating Sequentially Regulates Bone Regeneration to Enhance Interfacial Osseointegration

Wei Zhou[1,2#], Yang Liu[1#], Xuan Nie[3#], Chen Zhu[1,*], Liming Xiong[2,*], Jing Zhou[4,*] , Wei Huang[1,*]

Affiliations and addresses:

[1] Department of Orthopaedics, The First Affiliated Hospital of USTC, Division of Life Sciences and Medicine, University of Science and Technology of China, Hefei, China.

[2] Department of Orthopaedics, Union Hospital, Tongji Medical College, Huazhong University of Science and Technology, Wuhan, China.

[3] Department of Pharmacy, The First Affiliated Hospital of USTC, Division of Life Sciences and Medicine, Anhui Provincial Key Laboratory of Precision Pharmaceutical Preparations and Clinical Pharmacy, University of Science and Technology of China, Hefei, China.

[4] Department of Urology, The First Affiliated Hospital of USTC, Division of Life Sciences and Medicine, University of Science and Technology of China, Hefei, China.

***Corresponding Authors:** zhuchena@ustc.edu.cn (Chen Zhu); xiongliming@hust.edu.cn (Liming Xiong); jzhou21@ustc.edu.cn (Jing Zhou); zgkdhwei@ustc.edu.cn (Wei Huang).

[#]**These authors contributed equally:** Wei Zhou, Yang Liu, Xuan Nie.

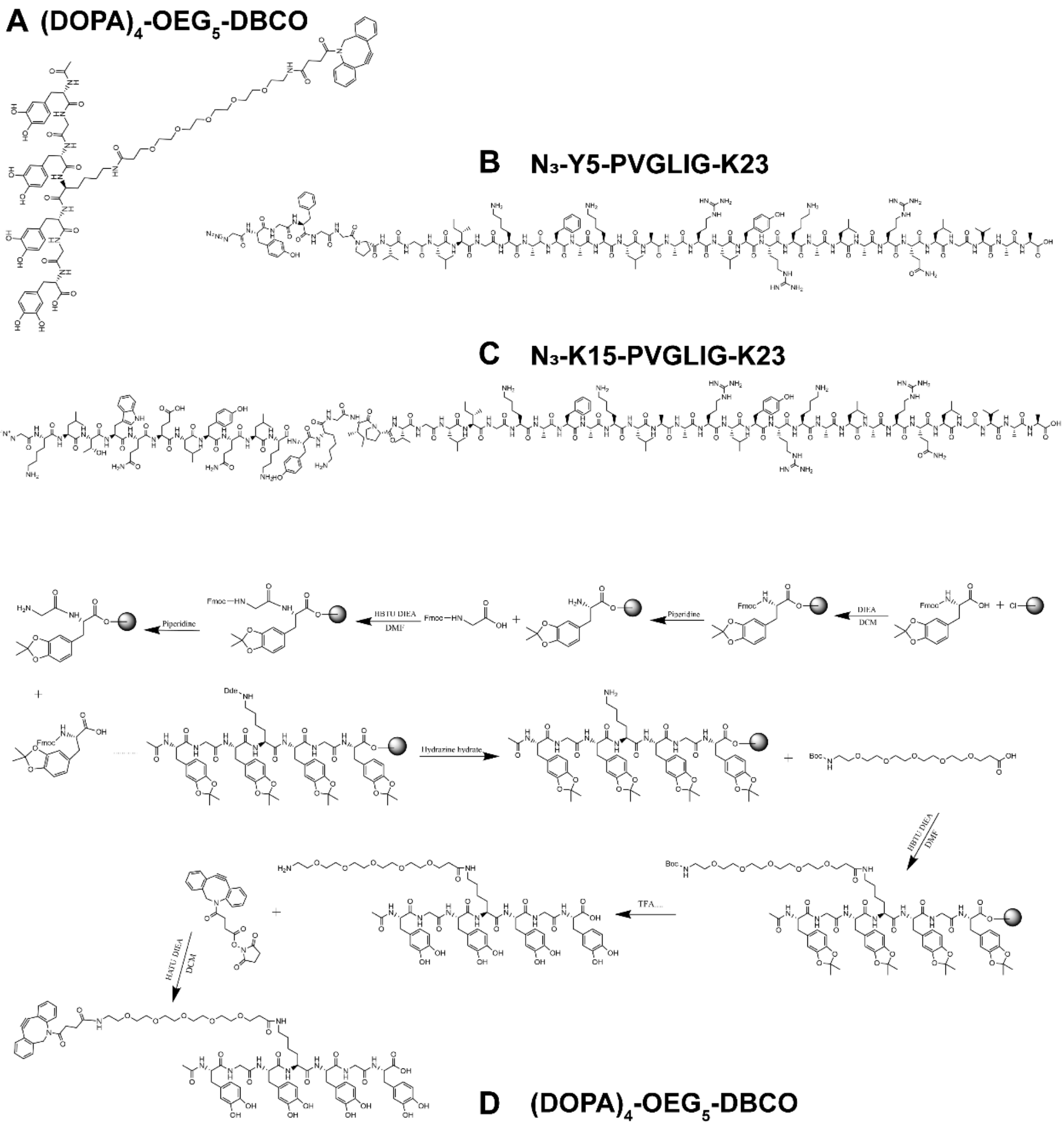

Supplementary Fig. 1 The molecular formula of the peptide and the synthesis process of $(DOPA)_4$-OEG_5-DBCO. (A–C) Molecular structures of $(DOPA)_4$-OEG_5-DBCO, P1 (N_3-K15-PVGLIG-K23), and P2 (N_3-Y5-PVGLIG-K23). **(D)** Synthesis process of $(DOPA)_4$-OEG_5-DBCO.

Supplementary Information

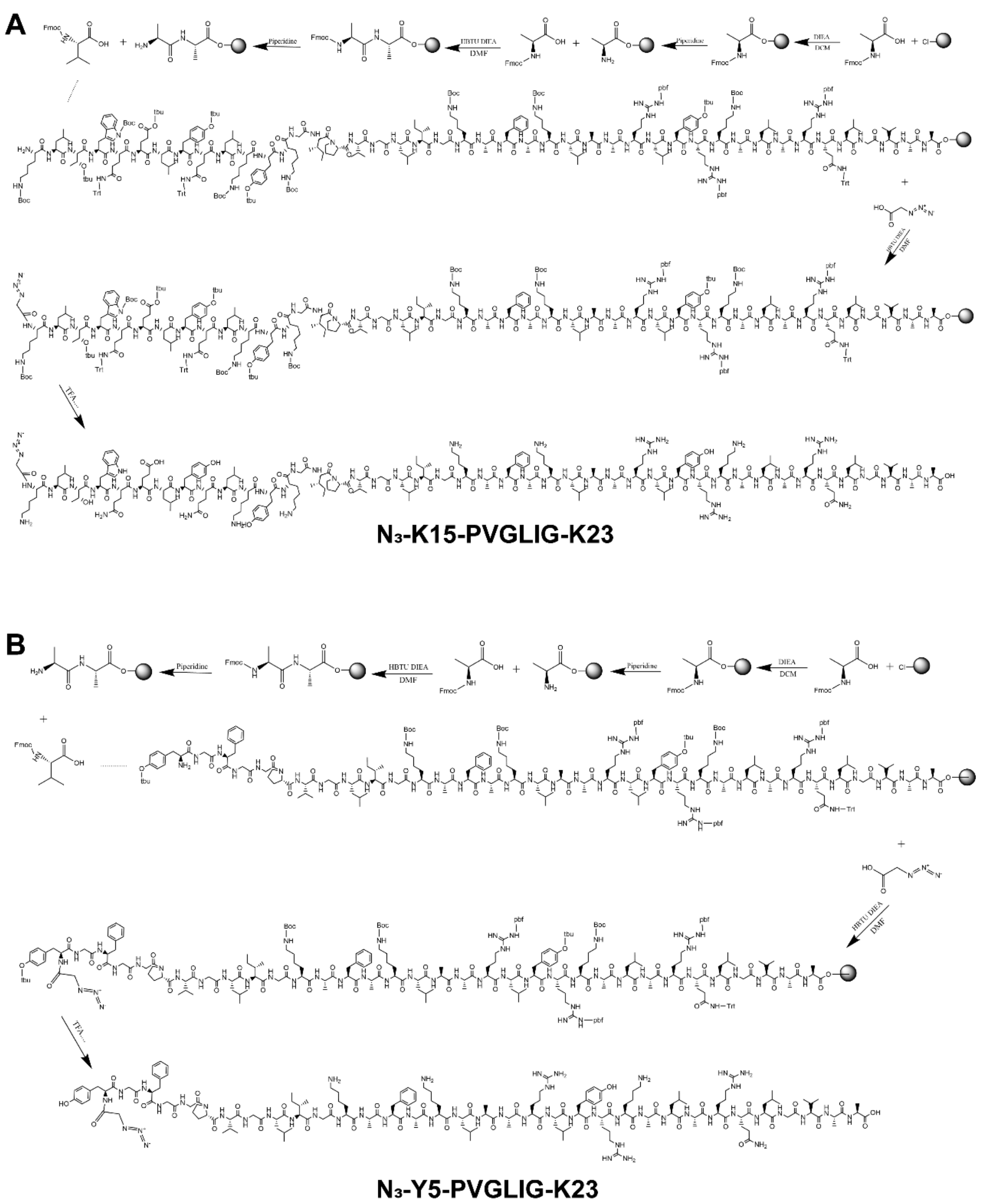

Supplementary Fig. 2 The synthesis process of P1 and P2. (A–B) Synthesis process of P1 (N_3-K15-PVGLIG-K23) and P2 (N_3-Y5-PVGLIG-K23).

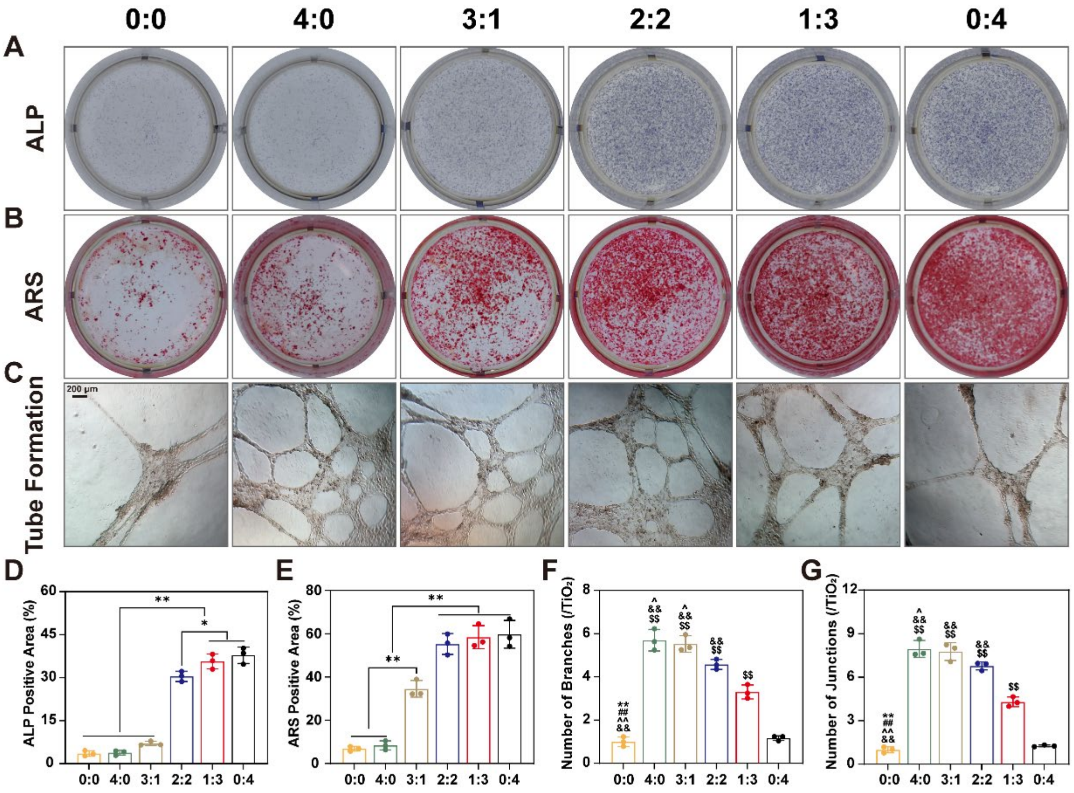

Supplementary Fig. 3 Exploration of the optimal ratio of P1 and P2. (A) Representative images of ALP staining and corresponding **(D)** quantitative analysis results after mixing P1 and P2 in different ratios (n=3 per group). **(B)** Representative images of ARS staining and corresponding **(E)** quantitative analysis results after mixing P1 and P2 in different ratios (n=3 per group). **(C)** Representative images of the tube formation assay and the corresponding **(F–G)** quantitative analysis results after mixing P1 and P2 in different ratios (n=3 per group). The data are presented as the mean ± standard deviation (SD). Data were analyzed by one-way ANOVA with Tukey's post hoc test ($*p < 0.05$, $**p < 0.01$ vs. the P1 : P2 = 4 : 0 group; $^{\#}p < 0.05$, $^{\#\#}p < 0.01$ vs. the P1 : P2 = 3 : 1 group; $^{\wedge}p < 0.05$, $^{\wedge\wedge}p < 0.01$ vs. the P1 : P2 = 2 : 2 group; $^{\&}p < 0.05$, $^{\&\&}p < 0.01$ vs. the P1 : P2 = 1 : 3 group; $^{\$}p < 0.05$, $^{\$\$}p < 0.01$ vs. the P1 : P2 = 0 : 4 group). $p < 0.05$ and $p < 0.01$ indicate statistical significance. Exact *p* values were given in the Source Data file.

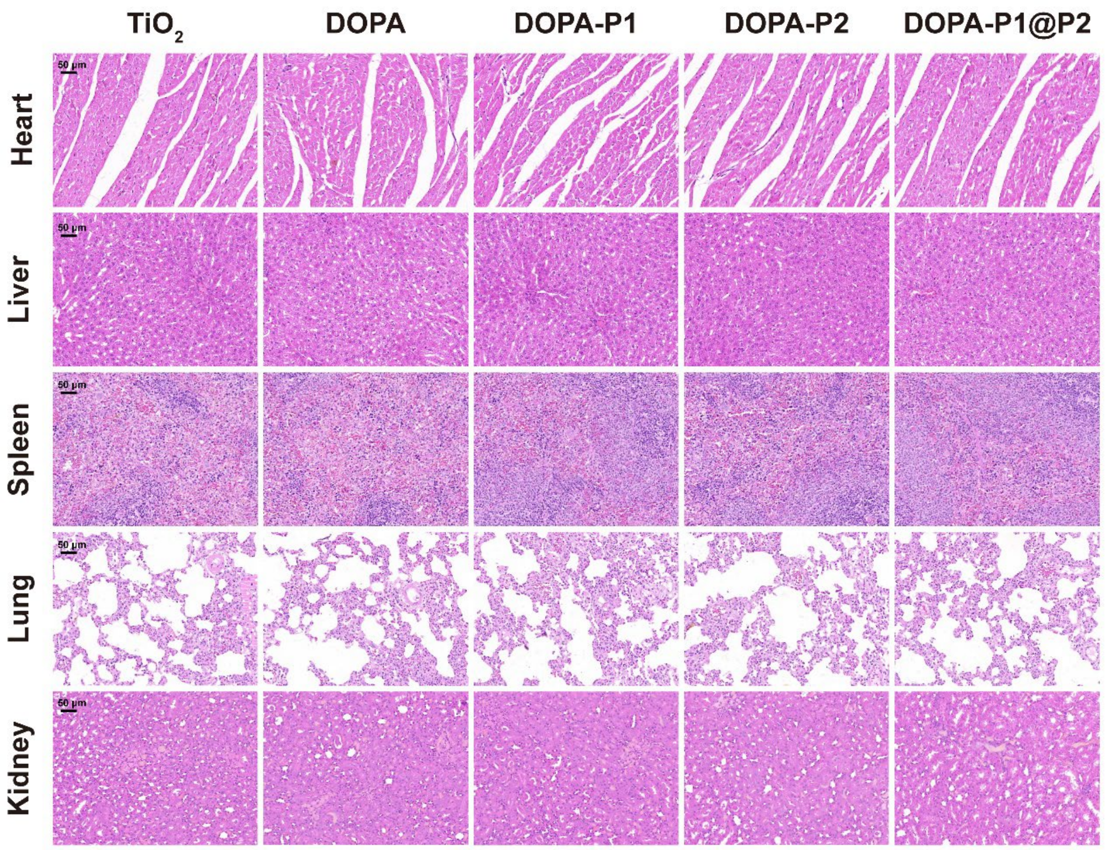

Supplementary Fig. 4: H&E staining of rat heart, liver, spleen, lung, and kidney at two months post-implantation.

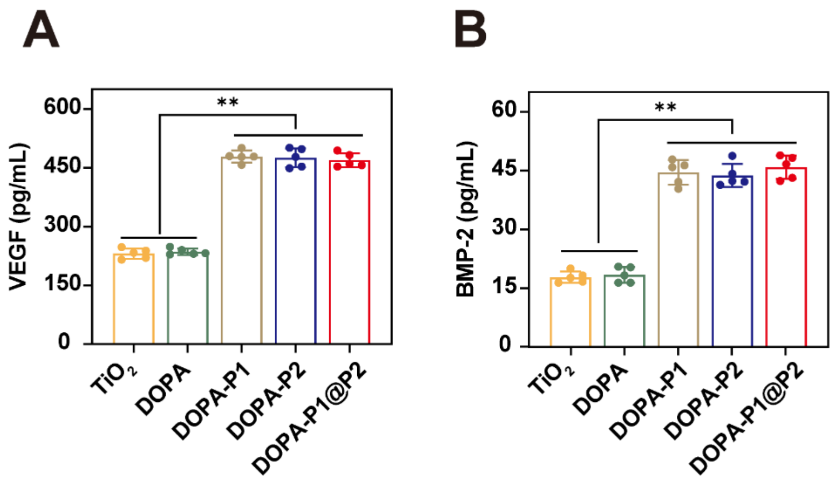

Supplementary Fig. 5 Macrophage M2 polarization secretes cytokines that promote angiogenesis and osteogenesis. (A–B) ELISA quantified the secretion levels of the angiogenesis-related protein VEGF and the osteogenesis-related protein BMP-2. The data are presented as the mean ± SD ; n = 5 per group. Data were analyzed by one-way ANOVA with Tukey's post hoc test, and **p* < 0.05 and ***p* < 0.01 indicate statistical significance. Exact *p* values were given in the Source Data file.

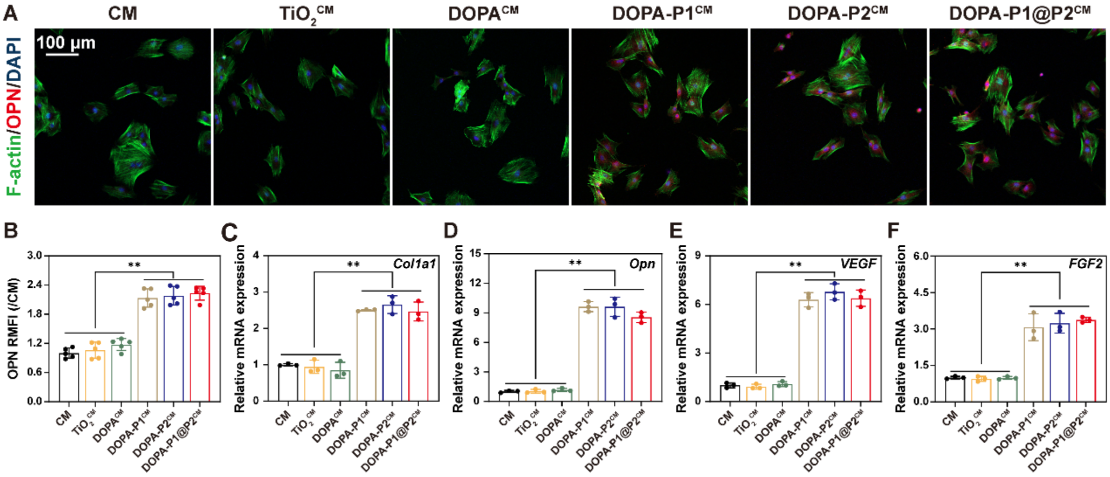

Supplementary Fig. 6 Supplementary experimental results on the indirect promotion of angiogenesis and osteogenesis. (A) Immunofluorescence staining evaluated the indirect osteogenic effects of modified surfaces, highlighting the cytoskeleton in green (F-actin), osteogenesis-related proteins in red (OPN), and nuclei in blue. **(B)** Quantitative results of immunofluorescence (n=5 per group). **(C–F)** RT-qPCR assessed the expression of osteogenic (*Colla1* and *Opn*) and angiogenic (*VEGF* and *FGF2*) genes to evaluate the indirect osteogenic effects of modified surfaces (n=3 per group). The data are presented as the mean ± SD. Data were analyzed by one-way ANOVA with Tukey's post hoc test, and $*p < 0.05$ and $**p < 0.01$ indicate statistical significance. Exact p values were given in the Source Data file.

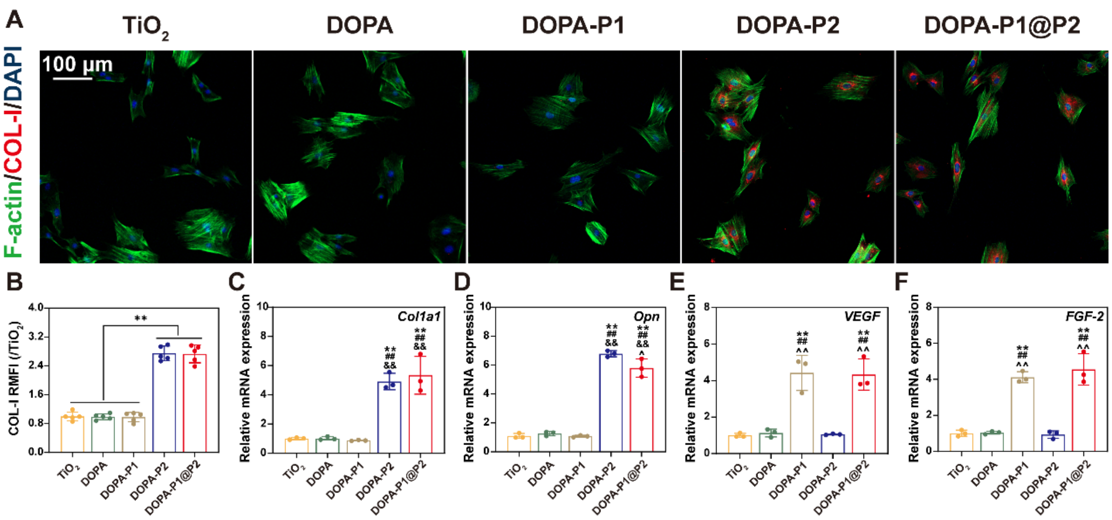

Supplementary Fig. 7 Supplementary experimental results on the direct promotion of angiogenesis and osteogenesis. (A) Immunofluorescence staining evaluated the direct osteogenic effects of modified surfaces, highlighting the cytoskeleton in green (F-actin), osteogenesis-related proteins in red (COL-I), and nuclei in blue. **(B)** Quantitative results of immunofluorescence (n=5 per group). **(C–F)** RT-qPCR assessed the expression of osteogenic (*Colla1* and *Opn*) and angiogenic (*VEGF* and *FGF2*) genes to evaluate the direct osteogenic effects of modified surfaces (n=3 per group). The data are presented as the mean ± SD. Data were analyzed by one-way ANOVA with Tukey's post hoc test ($^{*}p < 0.05$ and $^{**}p < 0.01$ vs. the TiO_2 group or comparisons between combined groups indicated in the figure; $^{\#}p < 0.05$ and $^{\#\#}p < 0.01$ vs. the DOPA group; $^{\&}p < 0.05$ and $^{\&\&}p < 0.01$ vs. the DOPA-P1 group; $^{\wedge}p < 0.05$ and $^{\wedge\wedge}p < 0.01$ vs. the DOPA-P2 group). $p < 0.05$ and $p < 0.01$ indicate statistical significance. Exact p values were given in the Source Data file.

Supplementary Information

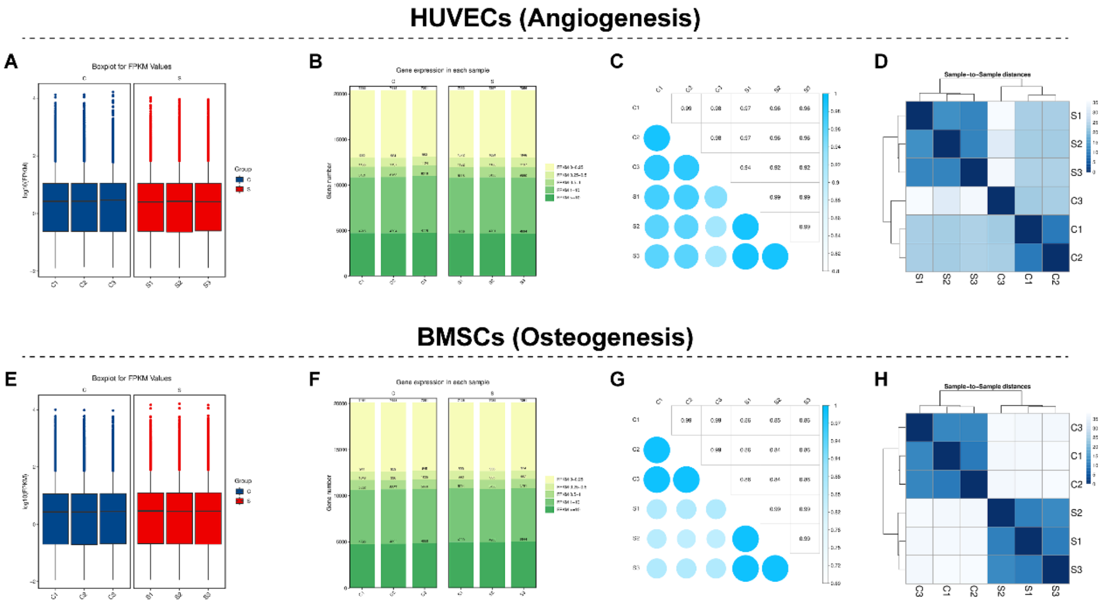

Supplementary Fig. 8 Supplementary quality control data for transcriptome sequencing. (A, E) Box plot of gene expression levels. **(B, F)** Area distribution map of gene expression levels. **(C, G)** Heatmap of correlation coefficients between samples. **(D, H)** Results of sample-to-sample cluster analysis. Statistical analysis was performed by unpaired two-tailed Student's *t*-test.

Supplementary Information

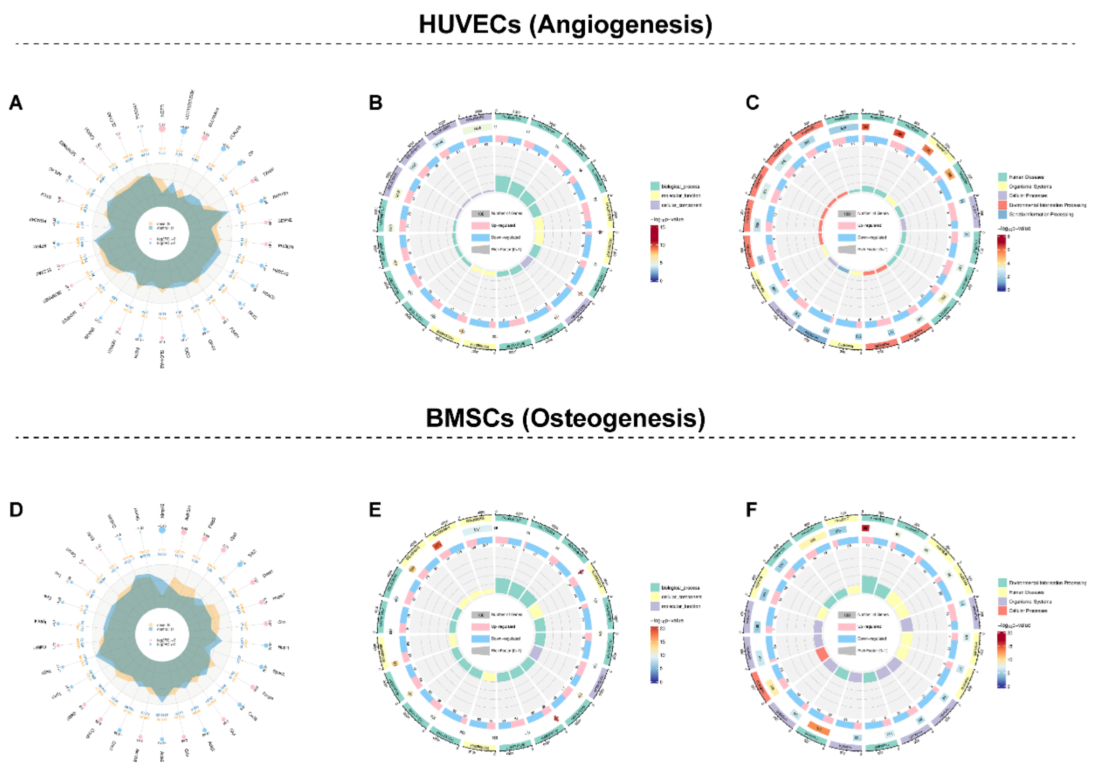

Supplementary Fig. 9 Supplementary GO and KEGG data for transcriptome sequencing. (A, D) Differential gene radar chart. **(B, E)** Circle plot of Gene Ontology (GO) enrichment analysis for differentially expressed genes. **(C, F)** Circle plot of Encyclopedia of Genes and Genomes (KEGG) enrichment analysis for differentially expressed genes. Statistical analysis was performed by unpaired two-tailed Student's *t*-test.

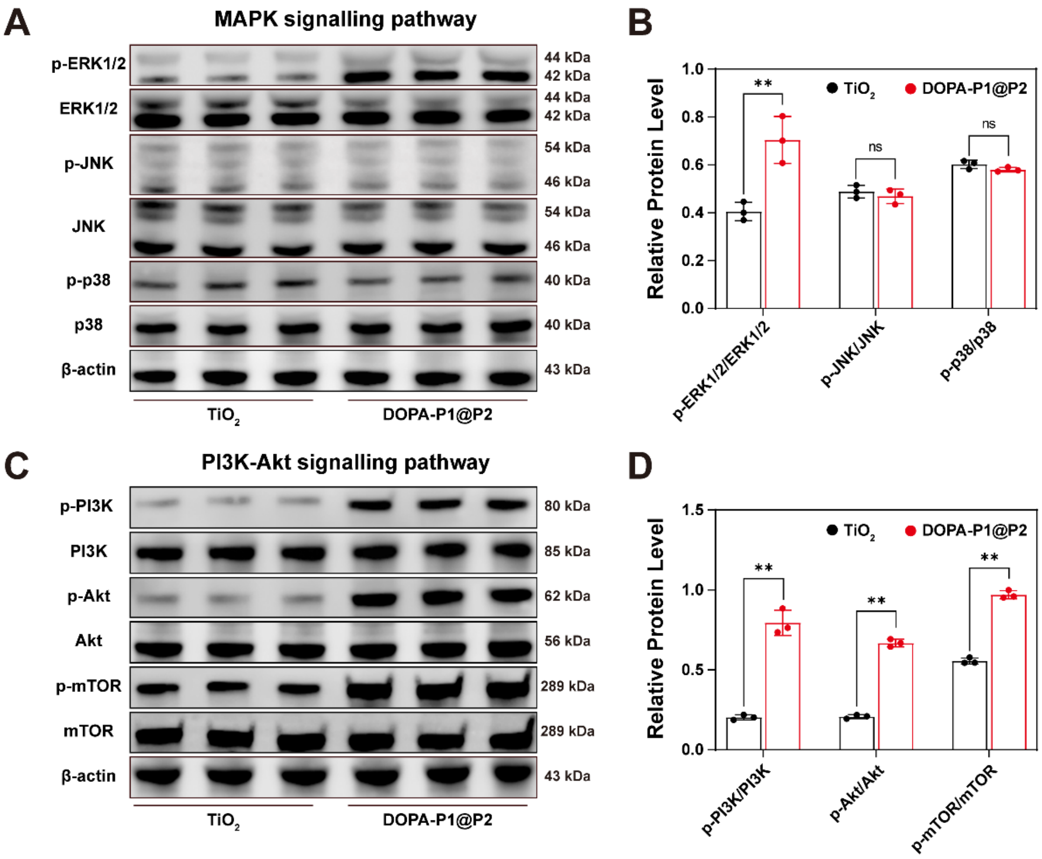

Supplementary Fig. 10 Validation of DOPA-P1@P2's mechanism in promoting angiogenesis and osteogenesis. (A–B) WB detected the expression levels of MAPK signalling pathway-related proteins, along with the corresponding quantitative analysis results (n=3 per group). **(C–D)** WB detected the expression levels of PI3K–AKT signalling pathway-related proteins, along with the corresponding quantitative analysis results (n=3 per group). The data are presented as the mean ± SD. Statistical analysis was performed by unpaired two-tailed Student's *t*-test., and $*p < 0.05$ and $**p < 0.01$ indicate statistical significance. Exact *p* values were given in the Source Data file.

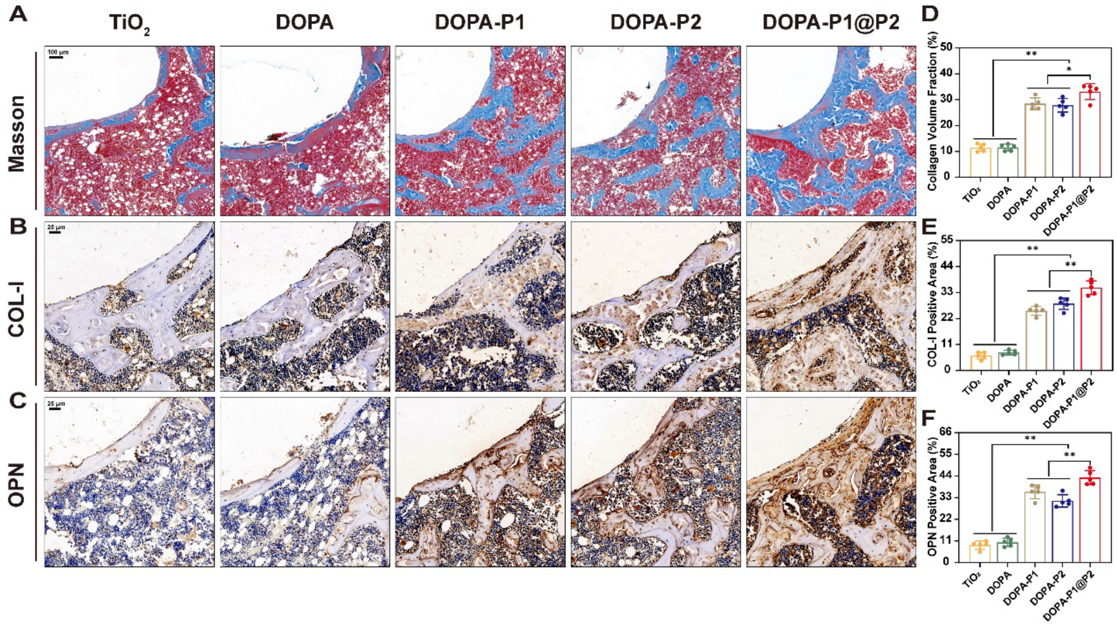

Supplementary Fig. 11: Supplementary in vivo experimental results on promoting osseointegration. (A, D) Masson's staining and corresponding quantitative analysis results (n=5 per group). **(B–C)** Immunohistochemical staining assessed the expression of osteogenic proteins (COL-I and OPN), accompanied by corresponding **(E–F)** quantitative analysis (n=5 per group). The data are presented as the mean ± SD. Data were analyzed by one-way ANOVA with Tukey's post hoc test, and $*p < 0.05$ and $**p < 0.01$ indicate statistical significance. Exact p values were given in the Source Data file.

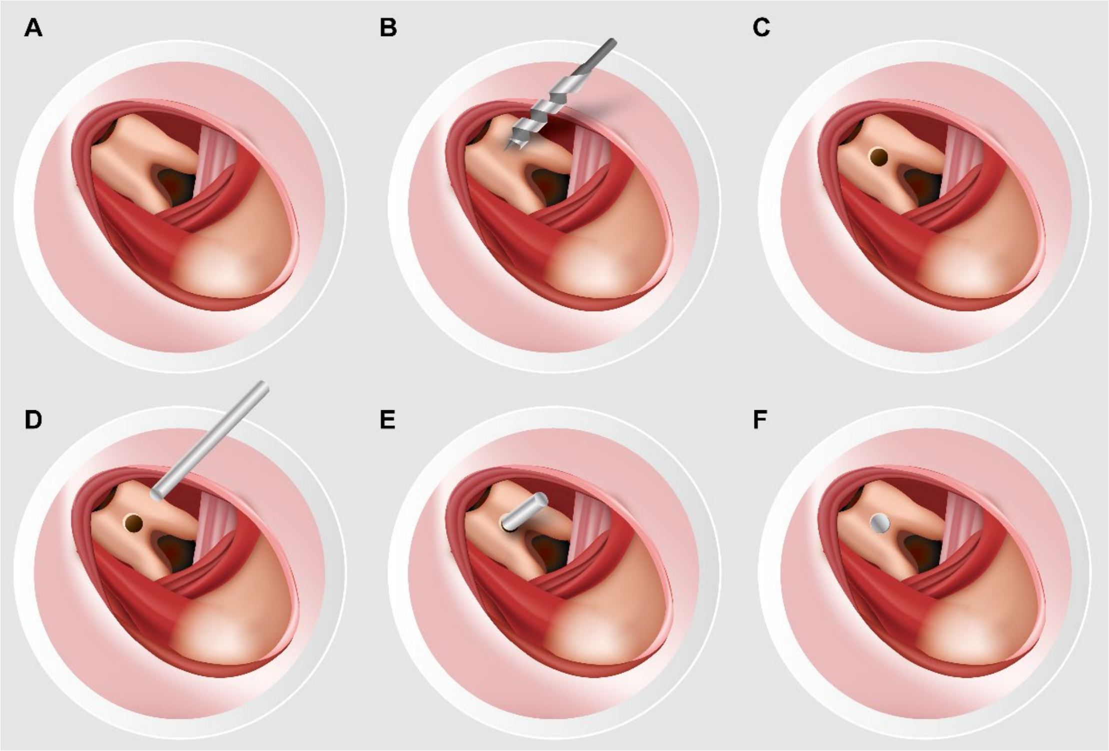

Supplementary Fig. 12 Schematic diagram of the animal model surgery. (A) Exposure of the femur condyles **(B)** Drilling of the femur condyles **(C)** Completion of the drilling process **(D–E)** Implantation of the titanium rod **(F)** Completion of the implantation process

Supplementary Information

Supplementary Table 1. Primer sequences used in RT-PCR analysis.

Genes	Primes (F-Forward, R-Reverse, 5'-3')
Mouse-*Tnf*	F: GTTCCCAAATGGCCTCCC<br>R: GTGCTCCTCACCCACACCG
Mouse-*Il6*	F: GAGACCACTGGGGAGAATGC<br>R: TTGCCAGGTGGGTAAAGTGG
Mouse-*Il10*	F: CCCTTTGCTATGGTGTCCT<br>R: GTGGCCAGTTTGTTATTTAT
Mouse-*Ccr7*	F: GGTGGCTCTCCTTGTCATTTTC<br>R: AGGTTGAGCAGGTAGGTATCCG
Mouse-*Cd206*	F: TACTTGGACGGATAGATGGAGG<br>R: CATAGAAAGGAATCCACGCAGT
Mouse-*Bmp2*	F: AACGAGAAAAGCGTCAAGCC<br>R: AGGTGCCACGATCCAGTCAT
Mouse-*Vegf*	F: AGGAGTCCCCGACGAGATAGA<br>R: CACATCTGCTGTGCTGTAGGAA
Mouse-*Actb*	F: GTGACGTTGACATCCGTAAAGA<br>R: GTAACAGTCCGCCTAGAAGCAC
Rat-*Col1a1*	F: AGCTCGATACACAATGGCCT<br>R: CCTATGACTTCTGCGTCTGG
Rat-*Opn*	F: GAACATGAAATGCTTCTTTCTCAG<br>R: TCCATGAAGCCACAAACTAAACTA
Rat-*Actb*	F: CCTCTATGACAACACAGT<br>R: AGCCACCAATCCACACAG
Human-*VEGF*	F: TATGCGGATCAAACCTCACCA<br>R: CACAGGGATTTTTCTTGTCTTGCT
Human-*FGF2*	F: AAGAGCGACCCTCACATCAA<br>R: GCCAGGTAACGGTTAGCACA
Human-*GADPH*	F: CATCATCCCTGCCTCTACTGG<br>R: GTGGGTGTCGCTGTTGAAGTC
