# Supplementary material for: Peptide-based inflammation-responsive implant coating sequentially regulates bone regeneration to enhance interfacial osseointegration
Source: Nat Commun. 2025 Apr 6;16:3283. doi: 10.1038/s41467-025-58444-8 (PMC11973180; doi:10.1038/s41467-025-58444-8)
Supplement: Supplementary file 2 — Reporting Summary [file 41467_2025_58444_MOESM2_ESM.pdf]

nature portfolio

Corresponding author(s): Chen Zhu, Liming Xiong, Jing Zhou,Wei Huang

Last updated by author(s): Feb 28, 2025

Reporting Summary

Nature Portfolio wishes to improve the reproducibility of the work that we publish. This form provides structure for consistency and transparency in reporting. For further information on Nature Portfolio policies, see our Editorial Policies and the Editorial Policy Checklist.

Statistics

For all statistical analyses, confirm that the following items are present in the figure legend, table legend, main text, or Methods section.

n/a	Confirmed	
☐	☒	The exact sample size (*n*) for each experimental group/condition, given as a discrete number and unit of measurement
☐	☒	A statement on whether measurements were taken from distinct samples or whether the same sample was measured repeatedly
☐	☒	The statistical test(s) used AND whether they are one- or two-sided<br>*Only common tests should be described solely by name; describe more complex techniques in the Methods section.*
☐	☒	A description of all covariates tested
☐	☒	A description of any assumptions or corrections, such as tests of normality and adjustment for multiple comparisons
☐	☒	A full description of the statistical parameters including central tendency (e.g. means) or other basic estimates (e.g. regression coefficient) AND variation (e.g. standard deviation) or associated estimates of uncertainty (e.g. confidence intervals)
☐	☒	For null hypothesis testing, the test statistic (e.g. *F*, *t*, *r*) with confidence intervals, effect sizes, degrees of freedom and *P* value noted<br>*Give P values as exact values whenever suitable.*
☒	☐	For Bayesian analysis, information on the choice of priors and Markov chain Monte Carlo settings
☒	☐	For hierarchical and complex designs, identification of the appropriate level for tests and full reporting of outcomes
☒	☐	Estimates of effect sizes (e.g. Cohen's *d*, Pearson's *r*), indicating how they were calculated

Our web collection on statistics for biologists contains articles on many of the points above.

Software and code

Policy information about availability of computer code

Data collection	All data needed to evaluate the conclusions in the paper are presented in the main text and supplementary materials. The RNA-seq data have been uploaded to the GEO and SRA (PRJNA1166748 and PRJNA1166738). You may view our study at https://dataview.ncbi.nlm.nih.gov/object/PRJNA1166748?reviewer=rdtj96efhb9r48gs5s9q5dckl9, and https://dataview.ncbi.nlm.nih.gov/object/PRJNA1166738?reviewer=l2t7t47u2n4b0oq54rauudlc93. Additional data related to this paper can be requested from the authors.
Data analysis	Code availability statement: No original code was generated for this project. The data analysis software used in this study are as follows: Originpro 2024, GraphPad Prism 10.1.0, ImageJ software (version 1.54d) and FlowJo™ software (version 10.4).

For manuscripts utilizing custom algorithms or software that are central to the research but not yet described in published literature, software must be made available to editors and reviewers. We strongly encourage code deposition in a community repository (e.g. GitHub). See the Nature Portfolio guidelines for submitting code & software for further information.

Data

Policy information about availability of data

All manuscripts must include a data availability statement. This statement should provide the following information, where applicable:

- Accession codes, unique identifiers, or web links for publicly available datasets
- A description of any restrictions on data availability
- For clinical datasets or third party data, please ensure that the statement adheres to our policy

Data availability statement:
All data supporting the conclusions of this paper are included in the main text and supplementary materials. Source data have been uploaded.

Research involving human participants, their data, or biological material

Policy information about studies with human participants or human data. See also policy information about sex, gender (identity/presentation), and sexual orientation and race, ethnicity and racism.

Reporting on sex and gender	n/a
Reporting on race, ethnicity, or other socially relevant groupings	n/a
Population characteristics	n/a
Recruitment	n/a
Ethics oversight	n/a

Note that full information on the approval of the study protocol must also be provided in the manuscript.

Field-specific reporting

Please select the one below that is the best fit for your research. If you are not sure, read the appropriate sections before making your selection.

☒ Life sciences ☐ Behavioural & social sciences ☐ Ecological, evolutionary & environmental sciences

For a reference copy of the document with all sections, see nature.com/documents/nr-reporting-summary-flat.pdf

Life sciences study design

All studies must disclose on these points even when the disclosure is negative.

Sample size	The sample sizes were estimated based on previous studies and have been listed in the manuscript.<br>At least 3 random samples were selected for statistical analysis in each experiment.
Data exclusions	No data were excluded
Replication	We are presenting one representative experiment out of at least three independent experiments performed.
Randomization	Each experiment was conducted in the form of random samples for statistical analysis and results presentation, including animal grouping.
Blinding	In the process of data collection and analysis, the researchers assigned all groups blindly. The data were anonymized for statistical analysis.

Reporting for specific materials, systems and methods

We require information from authors about some types of materials, experimental systems and methods used in many studies. Here, indicate whether each material, system or method listed is relevant to your study. If you are not sure if a list item applies to your research, read the appropriate section before selecting a response.

Materials & experimental systems

n/a	Involved in the study
☐	☒ Antibodies
☐	☒ Eukaryotic cell lines
☒	☐ Palaeontology and archaeology
☐	☒ Animals and other organisms
☒	☐ Clinical data
☒	☐ Dual use research of concern
☒	☐ Plants

Methods

n/a	Involved in the study
☒	☐ ChIP-seq
☐	☒ Flow cytometry
☒	☐ MRI-based neuroimaging

Antibodies

Antibodies used: iNOS (Abcam, ab178945), Arg-1 (Abcam, ab91279), VEGF (Immunoway, YN5444), FGF-2 (Abcam, ab208687), COL-I (Abcam, ab270993), OPN (Immunoway, YT3467), and β-actin (Immunoway, YM3028).

Validation: All the commercial antibodies have been validated by manufactures and the statements can be found on the manufactures' websites. Antibodies were all validated by positive control experiments.

Eukaryotic cell lines

Policy information about cell lines and Sex and Gender in Research

Cell line source(s): The BMSCs were isolated from the bone marrow of femur and tibia of 4-week-old Sprague Dawley rats. BMDMs were isolated from the bone marrow of 6-week-old C57BL/6 mice. HUVEC line were supplied by the Institute of Biochemistry and Cell Biology of the Chinese Academy of Sciences (Shanghai, China).

Authentication: Cell line were authenticated by the companies or the donors.

Mycoplasma contamination: All cell lines were tested for mycoplasma contamination. No mycoplasma contamination was found.

Commonly misidentified lines (See ICLAC register): No commonly misidentified lines were used.

Animals and other research organisms

Policy information about studies involving animals; ARRIVE guidelines recommended for reporting animal research, and Sex and Gender in Research

Laboratory animals: Sprague Dawley rats (4-week-old and 8-week-old); C57BL/6 mice (6-week-old)

Wild animals: No wild animals were used in this study.

Reporting on sex: All experimental models include both male and female rats.

Field-collected samples: No field-collected samples were used in the study.

Ethics oversight: All procedures involving animals strictly adhered to animal welfare guidelines and were approved by the Ethics Committee of Union Hospital, Tongji Medical College, Huazhong University of Science and Technology, with the corresponding approval number: [2022] IACUC Number: 3625.

Note that full information on the approval of the study protocol must also be provided in the manuscript.

Plants

Seed stocks: n/a

Novel plant genotypes: n/a

Authentication: n/a

Flow Cytometry

Plots

Confirm that:

- [x] The axis labels state the marker and fluorochrome used (e.g. CD4-FITC).
- [x] The axis scales are clearly visible. Include numbers along axes only for bottom left plot of group (a 'group' is an analysis of identical markers).
- [x] All plots are contour plots with outliers or pseudocolor plots.
- [x] A numerical value for number of cells or percentage (with statistics) is provided.

Methodology

Sample preparation	Cells from every group were collected and incubated for 30 minutes with antibodies specific for F4/80 (Thermo, 11-4801-82), CD206 (Thermo, 17-2061-80), and CD86 (Thermo, 12-0862-81). After rinsing the samples twice using PBS supplemented with 5% BSA, the cells were then resuspended. Finally, the subsets of M1 and M2 macrophages within the different samples were identified using flow cytometry
Instrument	Flow cytometry (Thermo Scientific, USA)
Software	All data were analyzed using FlowJo™ software (version 10.4).
Cell population abundance	BMSCs and BMDMs
Gating strategy	Gating strategy appear in Extended data of relevant Figures.

- [x] Tick this box to confirm that a figure exemplifying the gating strategy is provided in the Supplementary Information.
